# Supplementary material for: Generation of three-dimensional meat-like tissue from stable pig epiblast stem cells
Source: Nat Commun. 2023 Dec 9;14:8163. doi: 10.1038/s41467-023-44001-8 (PMC10710416; doi:10.1038/s41467-023-44001-8)
Supplement: Supplementary file 1 — Supplementary Information [file 41467_2023_44001_MOESM1_ESM.pdf]

Supplementary Information for

Generation of three-dimensional meat-like tissue from stable pig epiblast stem cells

Authors

Gaoxiang Zhu[1,5], Dengfeng Gao[1,5], Linzi Li[2,5], Yixuan Yao[1], Yingjie Wang[1], Minglei Zhi[1], Jinying Zhang[1], Xinze Chen[1], Qianqian Zhu[1], Jie Gao[1], Tianzhi Chen[1], Xiaowei Zhang[1], Tong Wang[1], Suying Cao[3], Aijin Ma[4], Xianchao Feng[2] and Jianyong Han[1]

Affiliations

[1]State Key Laboratory of Animal Biotech Breeding, College of Biological Sciences, China Agricultural University, Beijing, China.

[2]College of Food Science and Engineering, Northwest A&F University, Yangling, Shaanxi, China.

[3]Animal Science and Technology College, Beijing University of Agriculture, Beijing, China.

[4]School of Food and Health, Beijing Technology and Business University, Beijing, China.

[5]These authors contributed equally: Gaoxiang Zhu, Dengfeng Gao, Linzi Li.

Correspondence should be addressed to A.M. (email: maaj@btbu.edu.cn), X.F. (email: fengxianchao1@hotmail.com); J.H. (email: hanjy@cau.edu.cn).

The SI includes Supplementary Figures 1-12 and Supplementary Tables 1-2.

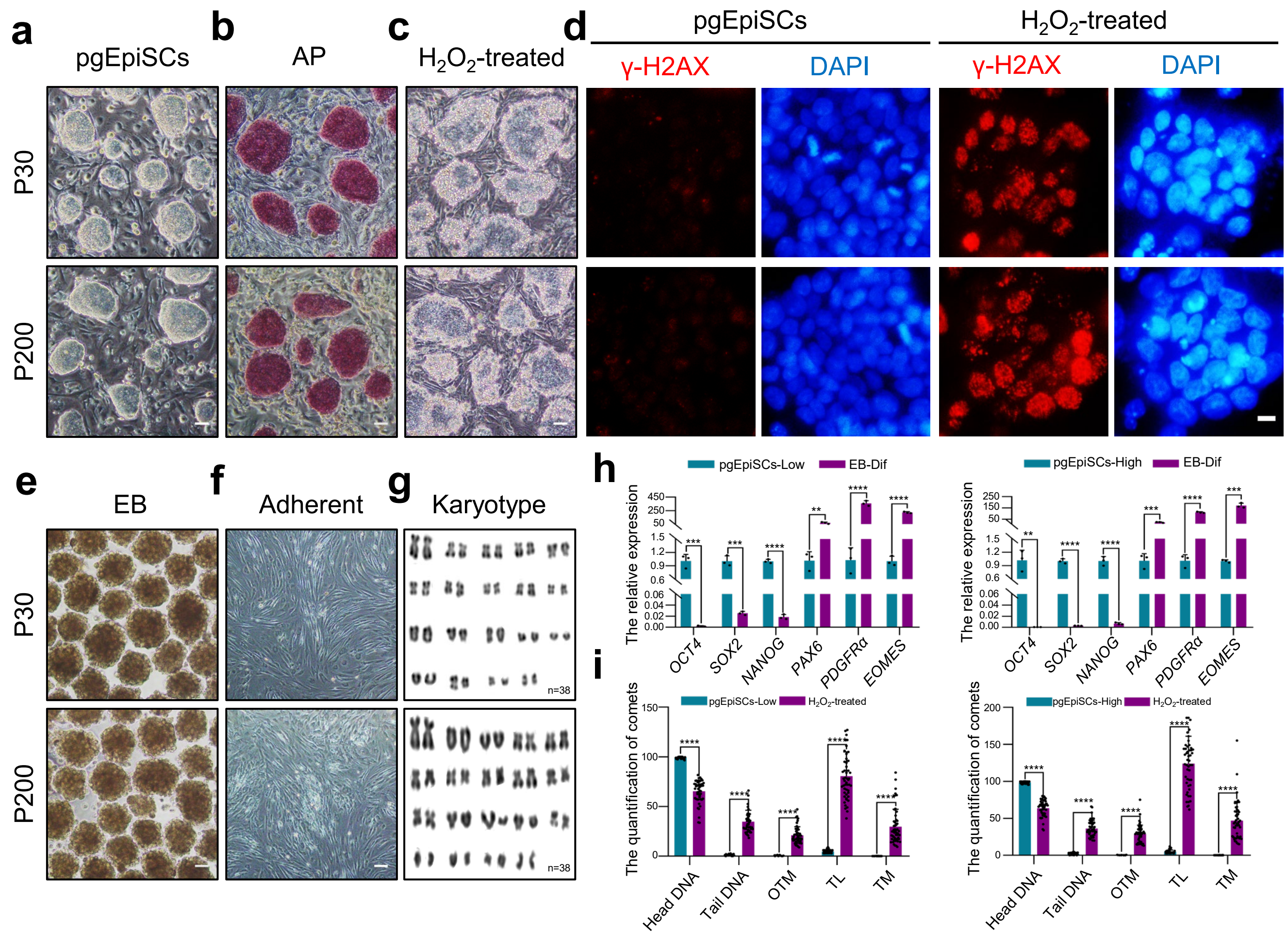

Supplementary Fig. 1: The pluripotency, differentiation potential and genomic stability of pgEpiSCs are not affected by increasing passage (related to Fig. 1). a Observations on the morphology of pgEpiSCs at low and high passages. Scale bar, 50 μm. **b** Comparison of alkaline phosphatase activities of pgEpiSCs at low and high passages. Scale bar, 50 μm. **c** Morphology of pgEpiSCs at low and high passages after H_2O_2 treatment. Scale bar, 50 μm. **d** Immunofluorescence staining of γ-H2AX of pgEpiSCs at low and high passages; H_2O_2 treatment was used as a positive control; DAPI was used for nucleus staining. Scale bar, 10μm. **e-f**, Morphological observation of EB spheres and random differentiated adnexal cells of pgEpiSCs at low and high passages. Scale bar, 50 μm. **g** Karyotype analysis of pgEpiSCs at low and high passages (top: P30, bottom: P200), which maintain normal chromosome numbers (n=38). **h** The expression of relevant genes related to pluripotency (*OCT4*, *SOX2*, *NANOG*), ectoderm (*PAX6*), mesoderm (*PDGFRα*), and endoderm (*EOMES*) at low and high passages of pgEpiSCs of EB random differentiation, n = 3, EB-Dif: EB differentiation. **i** Quantification of the extent of comets between low and high passages of pgEpiSCs and H_2O_2-treated groups (n = 50 nuclei) is shown; OTM: olive tail moment, TL: tail length, TM: tail moment. For **h** and **i**, error bars indicate means ± SD; ** $p < 0.01$, *** $p < 0.001$, **** $p < 0.0001$. Represent significant using two-tailed student's t test and similar results were obtained in three independent experiments. Exact *P* values are listed in Source Data Fig S1. For **a-h**, Low: P30; High: P200. For **i**, Low: P24; High: P194.

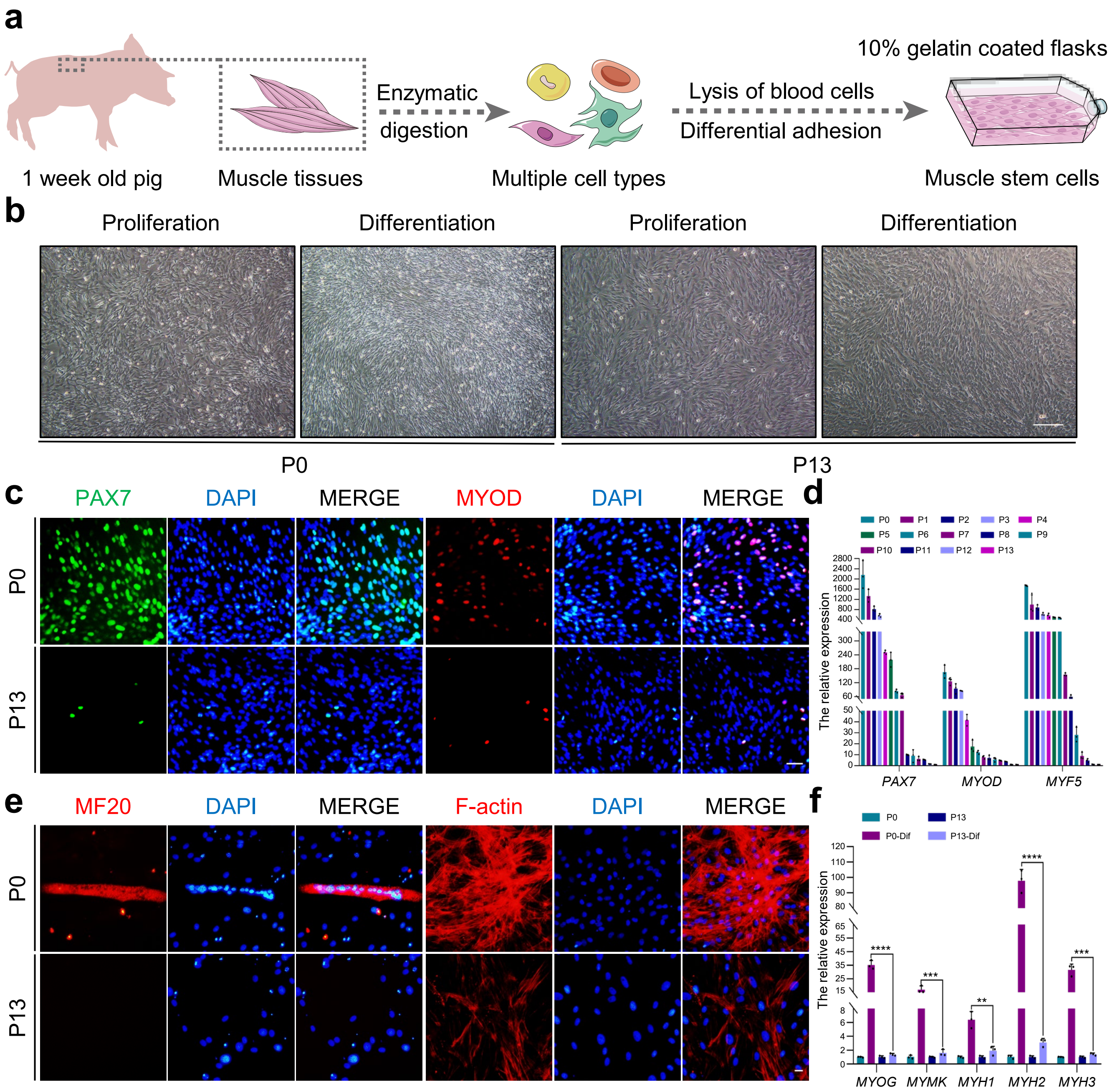

Supplementary Fig. 2: Porcine muscle stem cells (pMuSCs) attenuate stemness after long-term expansion *in vitro* (related to Fig. 1). a Schematic diagram of pMuSCs isolation. **b** Morphology of proliferation and differentiation of pMuSCs for different passages. Scale bar, 200 μm. **c** Immunofluorescent staining of PAX7 (green) and MYOD (red) for different passages of pMuSCs. Blue indicated DAPI. Scale bar, 20 μm. **d** qRT-PCR analysis of *PAX7*, *MYOD*, and *MYF5* mRNA levels from cells being expanded for different passages. **e** Immunofluorescent staining of MF20 (red) and F-actin (red) of myotubes differentiated from P0 and P13; DAPI was used for nucleus staining. Scale bar, 20 μm. **f** Expression of *MYOG*, *MYMK*, *MYH1*, *MYH2*, and *MYH3* of myotubes differentiated from P0 and P13. For **d** and **f**, error bars indicate means ± SD, ** $p < 0.01$, *** $p < 0.001$, **** $p < 0.0001$. Represent significant using two-tailed student's t test and similar results were obtained in three independent experiments. Exact *P* values are listed in Source Data Fig S2.

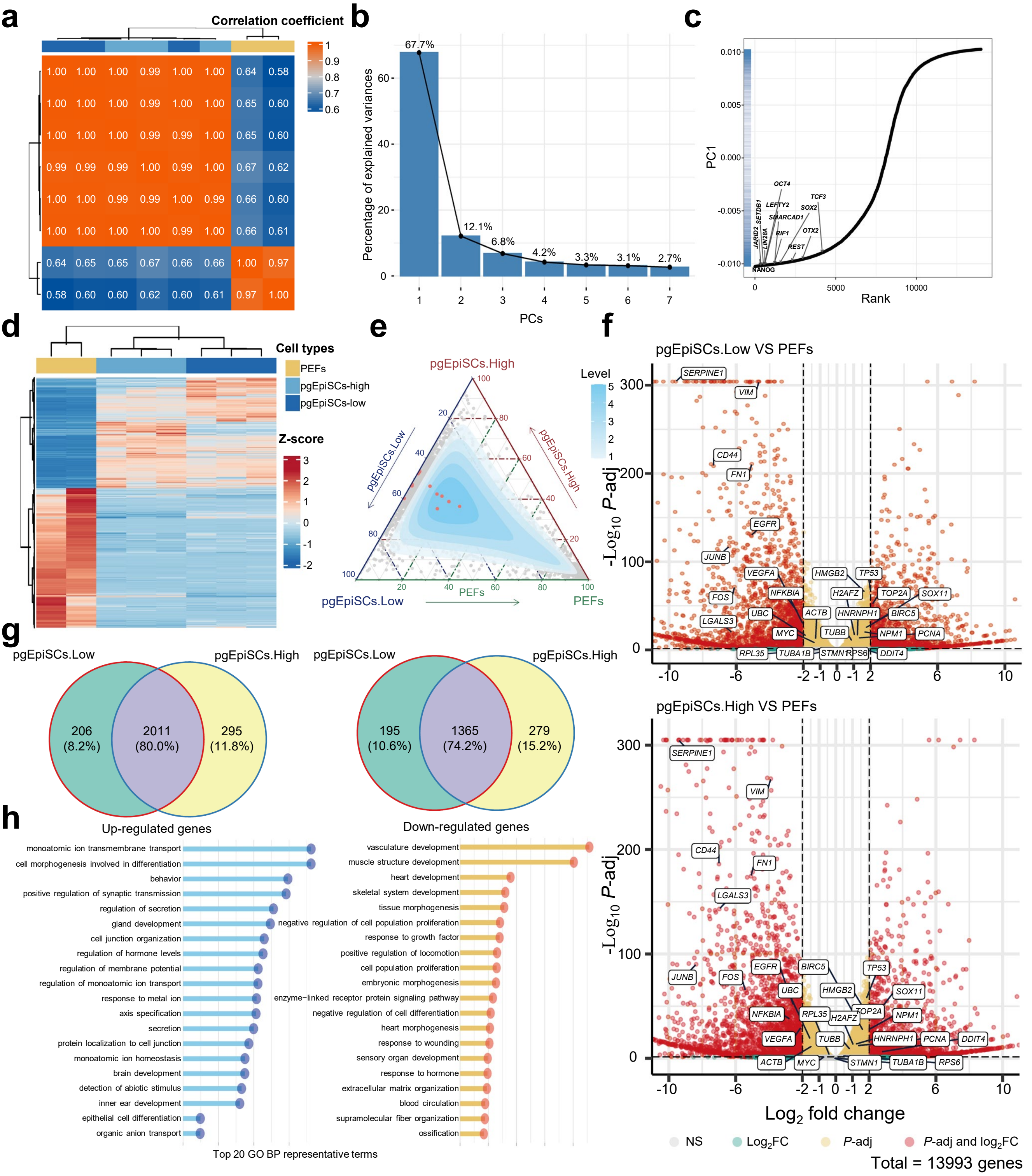

Supplementary Fig. 3: Transcriptome analysis of pgEpiSCs at low and high passages (related to Fig. 1). a Pearson's correlation coefficients between PEF and low, high passages of pgEpiSCs. **b** The bar plot of eigenvalues corresponds to the amount of variation explained by each principal component (PC) between low and high passages of pgEpiSCs, and PEFs. **c** Loading scores of the genes for PC1 of pgEpiSCs at low and high passages. **d** Heatmap of total DEGs for pluripotency of pgEpiSCs at low and high passages, and PEFs. **e** Ternary plot of total expression genes for pluripotency of pgEpiSCs between low and high passages, and PEFs. Key pluripotency markers were highlighted in red. **f** Volcano plot of differentially expressed genes between pgEpiSCs and PEFs. Significant genes are in red. Cancer-associated hallmark genes (TP53) are not different expressed (labeld with white box). The Wald test of the DESeq2 R package was applied for differential gene analysis. The Benjamini-Hochberg adjusted *P*-value (*P*-adj) < 0.05 and absolute $\log2$ (fold change) >2 are cut-offs for statistical significance. **g** Venn of DEGs (left: up-regulated, right: down-regulated) between PEFs and pgEpiSCs at low and high passages. **h** GO-BP enrichment analysis of DEGs (left: up-regulated, right: down-regulated) between PEFs and pgEpiSCs at different passages. For **a**-**h**, Low: P30; High: P200.

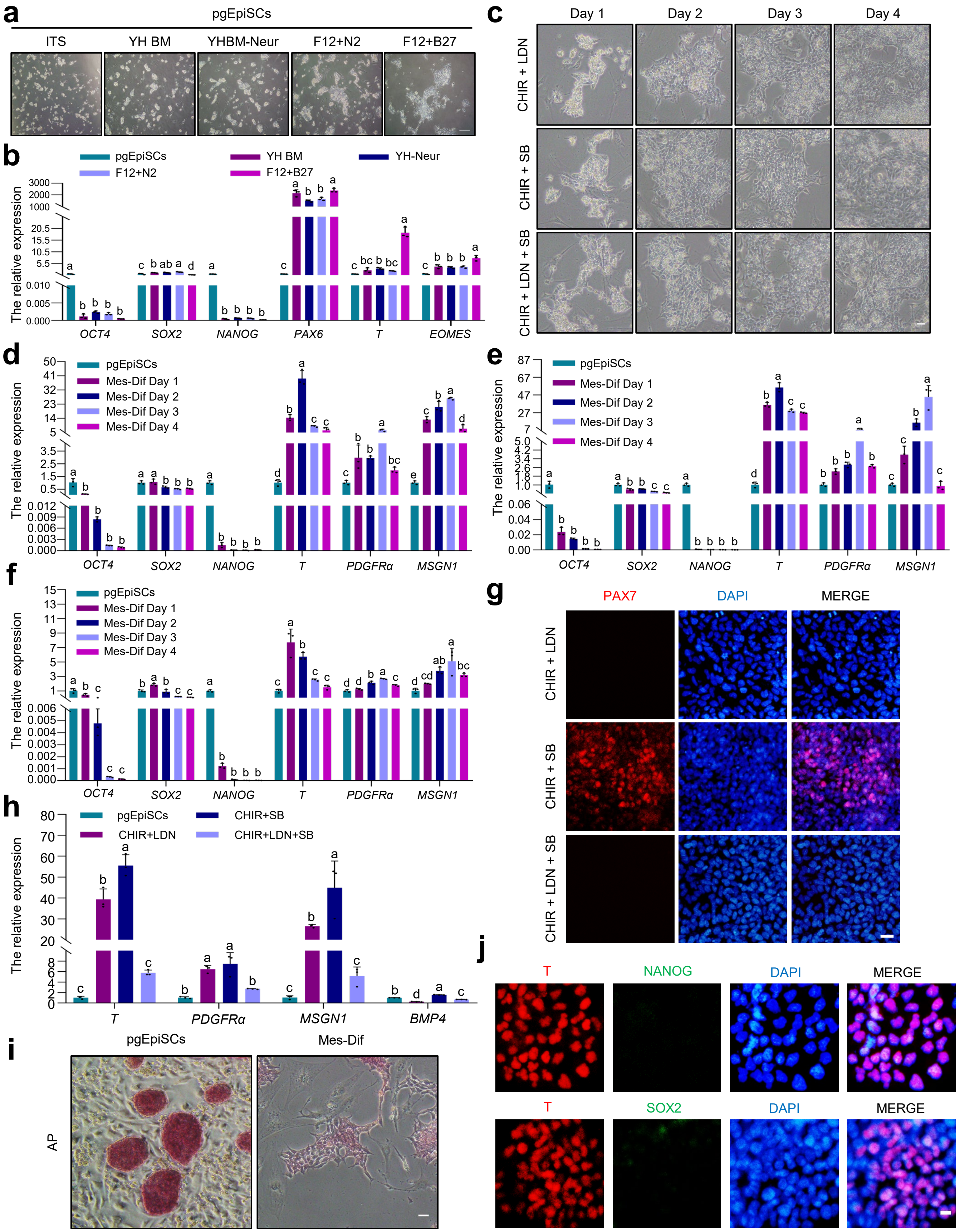

Supplementary Fig. 4: The small molecule screening of pgEpiSCs in the early stage of myogenic differentiation (related to Fig. 2). a Morphological observation of the appearance of cell adhesion in different systems without the addition of small molecules. Scale bar, 200 μm. **b** Expression of *OCT4*, *SOX2*, *NANOG*, *PAX6*, *T*, and *EOMES* for random differentiation in different systems. **c** Observation of the morphology of cell apposition and differentiation under different combinations of small molecules in MDM I. Scale bar, 50 μm. **d-f** Expression of genes related to pluripotency (*OCT4*, *SOX2*, *NANOG*) and paraxial mesodermal differentiation (*T*, *PDGFRα* and *MSGN1*) under the activation of WNT and the inhibition of BMP (**d**), the activation of WNT and the inhibition of TGF-β (**e**), or the activation of WNT and the inhibition of TGF-β and BMP (**f**) conditions. **g** Immunostaining of PAX7 in cells treated with different combinations of

small molecules in MDM I and continuously throughout differentiation to MDM III. DAPI was used for nuclear staining. Scale bar, 20 μm. **h** Expression of *T* (Day 2), *PDGFRα* (Day 3), *MSGN1* (Day 3) and *BMP4* (Day 3) under WNT activation and BMP inhibition, WNT activation and TGF-β inhibition, or WNT activation and TGF-β and BMP inhibition conditions. **i** Alkaline phosphatase staining under the activation of WNT and the inhibition of TGF-β conditions. Scale bar, 50 μm. **j** Immunostaining of T, NANOG and SOX2 in the presence of WNT activation and TGF-β inhibition; DAPI was used for nucleus staining. Scale bar, 10 μm. For **a** and **b**, ITS: insulin-transferrin-selenium; YH BM: basal medium for pgEpiSCs without the addition of any small molecules or growth factors; YH BM-Neur: the neurobasal was withdrawn from the basal medium of pgEpiSCs; F12+N2: the neurobasal and B27 were withdrawn from the basal medium of pgEpiSCs; F12+B27: the neurobasal and N2 were withdrawn from the basal medium of pgEpiSCs. For **c** and **g**, CHIR: CHIR99021; LDN: LDN193189; SB: SB431542. For **b**, **d**, **e**, **f** and **h**, error bars indicate means ± SD, n = 3. Data was analyzed using a one-way ANOVA, followed by Duncan's new multiple range test and different letters represent significant differences at $p < 0.05$. Similar results were obtained in three independent experiments. Mes-Dif: paraxial mesodermal differentiation. Exact *P* values are listed in Source Data Fig S4.

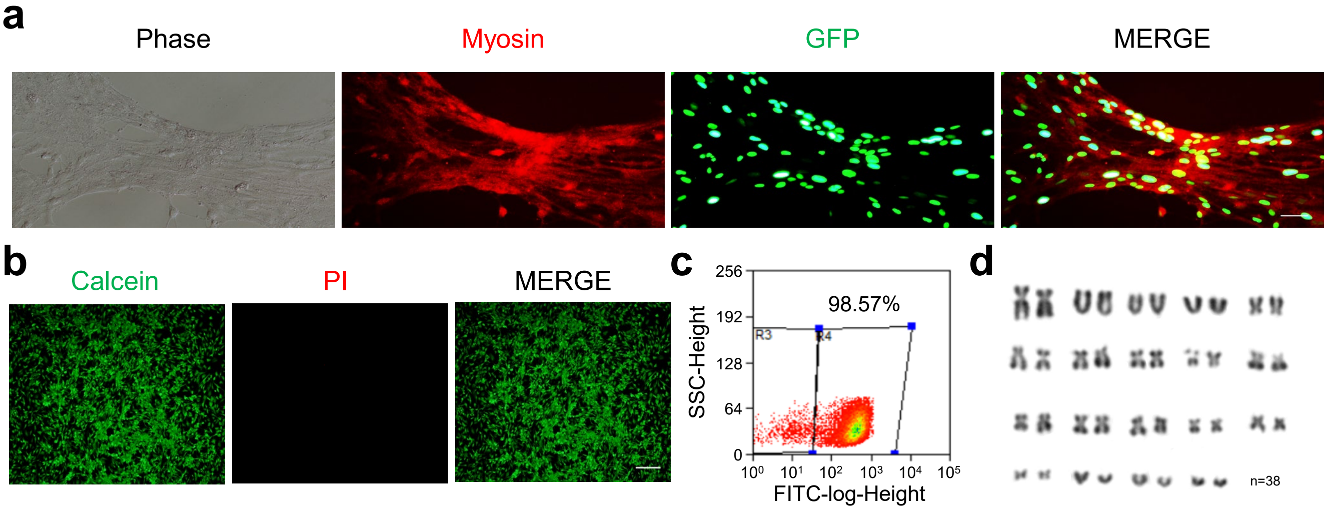

Supplementary Fig. 5: Identification of skeletal muscle fibers derived from pgEpiSCs (related to Fig. 2). a Myogenic differentiation was observed by using the pgEpiSCs-NLS-GFP cell line. The GFP represents the nucleus and can be observed in single myofibers possessing multiple nuclear fusions. Scale bar, 50 μm. **b** Representative images of Calcein-AM (live, green) and PI (dead, red) of pgEpiSCs-derived skeletal muscle fibers. Scale bar, 200 μm. **c** Flow cytometric assay for survival of pgEpiSCs-derived skeletal muscle fibers. **d** Karyotype analysis of pgEpiSCs after myogenic differentiation, which maintains normal chromosome numbers (n=38).

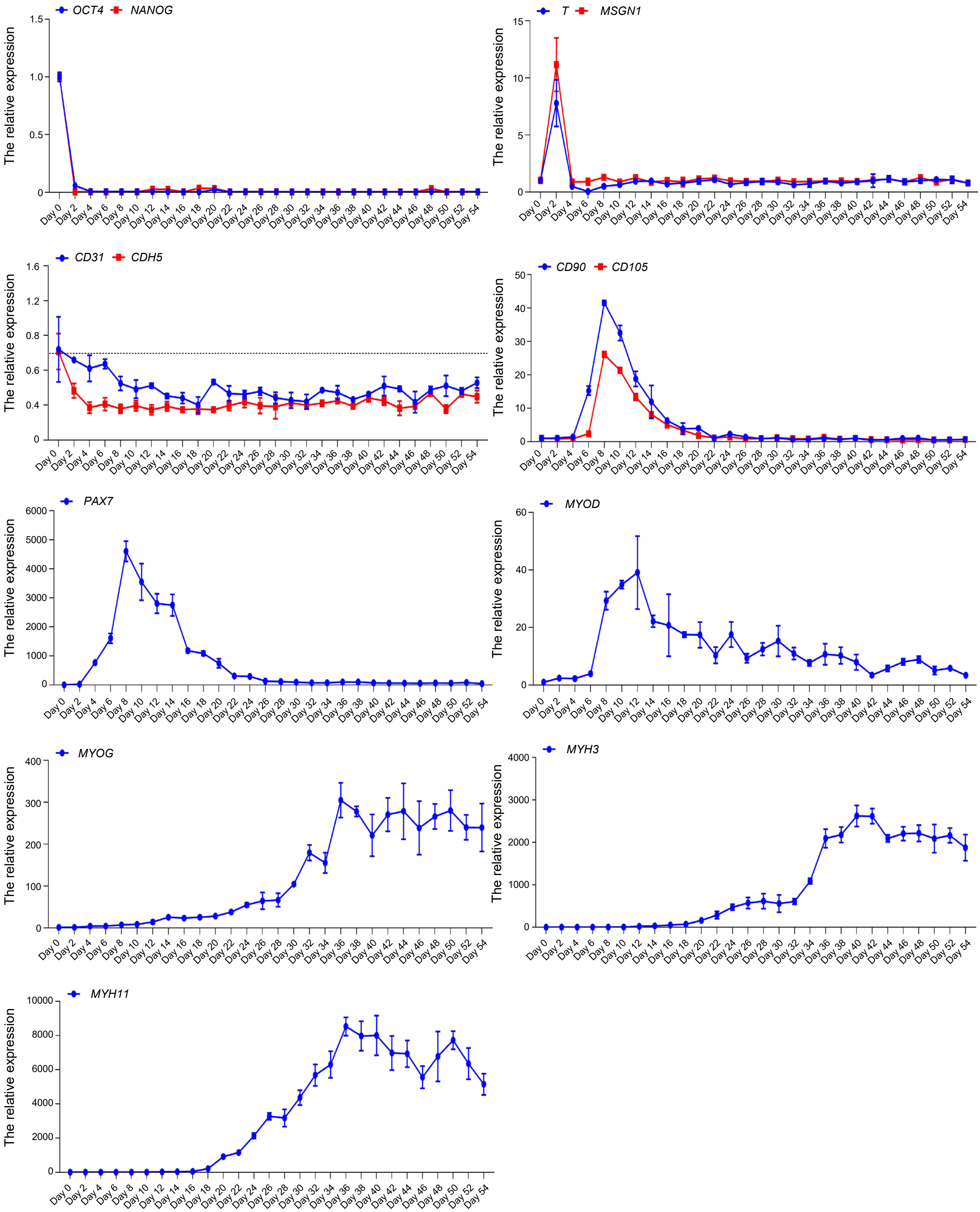

Supplementary Fig. 6: The detection of gene expression in pgEpiSCs during myogenic differentiation. (related to Fig. 2). The expression of relevant genes, including key marker genes related to pluripotency (*OCT4* and *NANOG*), paraxial mesoderm differentiation (*T* and *MSGN1*), muscle stem cells (*PAX7* and *MYOD*), myogenic maturation (*MYOG*, *MYH3*, and *MYH11*), mesenchymal differentiation (*CD90* and *CD105*), and endothelial differentiation (*CD31* and *CDH5*). Error bars indicate means ± SD and data are listed in Source Data Fig S6.

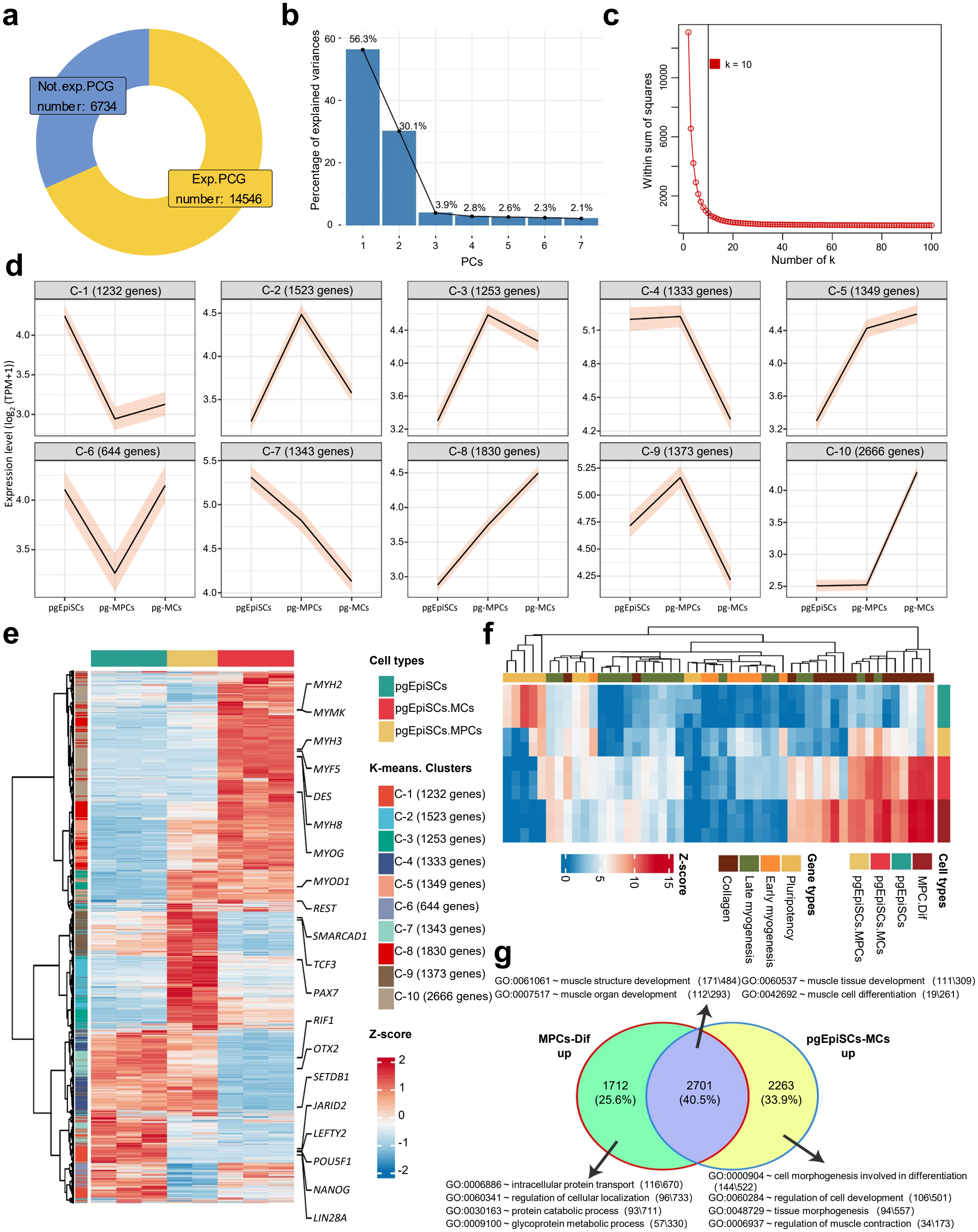

Supplementary Fig. 7: Ten clustering patterns from RNA-seq data analysis for myogenic differentiation of pgEpiSCs (related to Fig. 3). a The ratio of expressed and not expressed genes during the myogenesis of pgEpiSCs *in vitro*. **b** The barplot of eigenvalues corresponds to the amount of variation explained by each PC during the myogenesis of pgEpiSCs *in vitro*. **c** Scatter plots of values with different k values. **d** A total of 10 clustering categories were created by gene expression patterns in each stage of the pgEpiSCs, pgEpiSCs-MPCs and pgEpiSCs-MCs populations. **e** Heatmap of representative clusters for pluripotency and myogenic differentiation of pgEpiSCs, pgEpiSCs-MPCs and pgEpiSCs-MCs. **f** Heatmap of DEGs between differentiation of pMuSCs isolated *in vivo* and pgEpiSCs-derived MPCs and MCs. **g** Venn diagram of the upregulated genes and their GO terms in MCs from pMuSCs or pgEpiSCs. For **d**, **e**, **f** and **g**, pgEpiSCs-MPCs: pgEpiSCs-derived myogenic progenitor cells; pgEpiSCs-MCs: mature muscle fiber cells after N2 treatment; MPCs-Dif: mature myofibroblasts of pMuSCs after differentiation under 2% HS treatment.

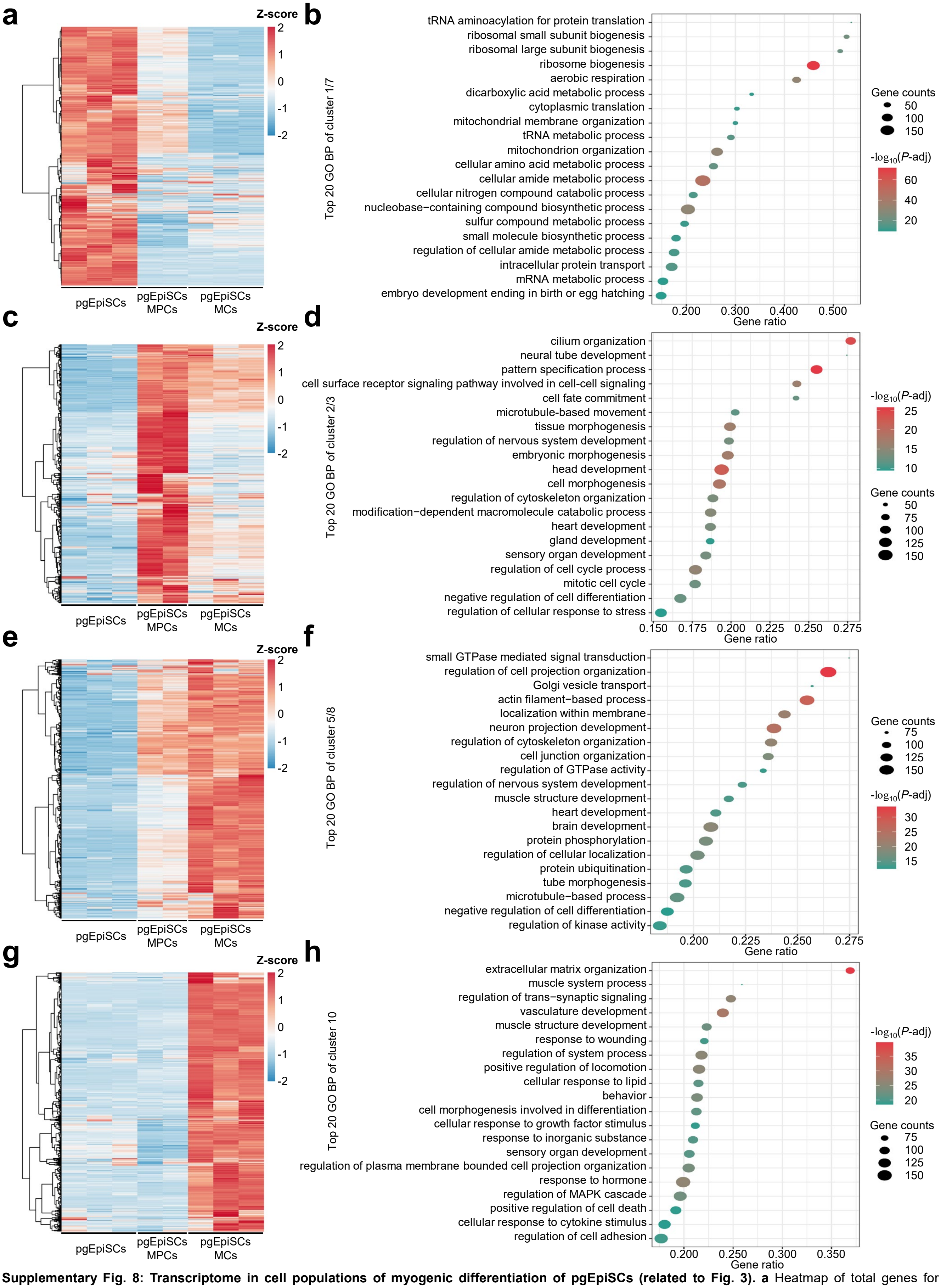

Supplementary Fig. 8: Transcriptome in cell populations of myogenic differentiation of pgEpiSCs (related to Fig. 3). **a** Heatmap of total genes for cluster 1 and 7. **b** Enriched GO terms with genes in clusters 1 and 7 from RNA-seq data. **c** Heatmap of total genes for clusters 2 and 3. **d** Enriched GO terms with genes in clusters 2 and 3 from RNA-seq data. **e** Heatmap of total genes for clusters 5 and 8. **f** Enriched GO terms with genes in clusters 5 and 8 from RNA-seq data. **g** Heatmap of total genes in cluster 10. **h** Enriched GO terms with genes of cluster 10 from RNA-seq data.

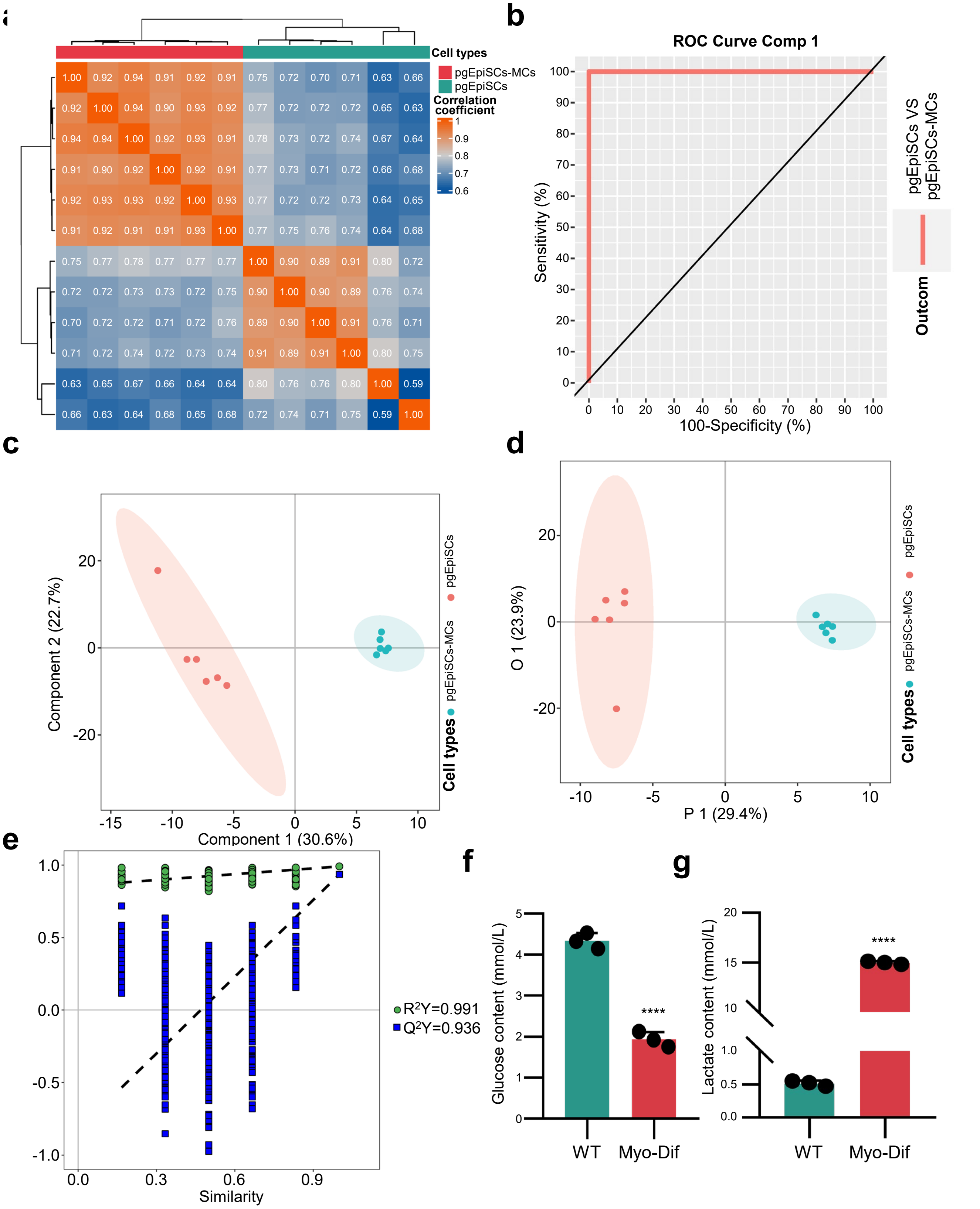

Supplementary Fig. 9: Data quality assessment of pgEpiSCs and pgEpiSCs-MCs metabolomes (related to Fig. 4). a Spearman's correlation coefficients between pgEpiSCs and pgEpiSCs-MCs. **b** ROC curve of partial least squares-discriminate analysis (PLSDA) result. The closer the area under the curve is to 1, the higher the reliability of the model. **c** PLSDA of pgEpiSCs and pgEpiSCs-MCs. **d** Orthogonal projections to latent structures discriminant analysis (OPLSDA) of pgEpiSCs and pgEpiSCs-MCs. **e** The cross-validation of the OPLSDA. R2 is the explainability and Q2 is the predictability. The predictability is measured by Q2, and the explainability by R2. The model performs better the closer the two are near 1. **f-g** Detection of glucose and lactate content in pgEpiSCs after myogenic differentiation. Error bars indicate means ± SD (n = 3), **** *p* < 0.0001. Represent significant using two-tailed student's t test and similar results were obtained in three independent experiments. Exact *P* values are listed in Source Data Fig S9. WT: undifferentiated pgEpiSCs, Myo-Dif: terminally differentiated cells after N2 treatment.

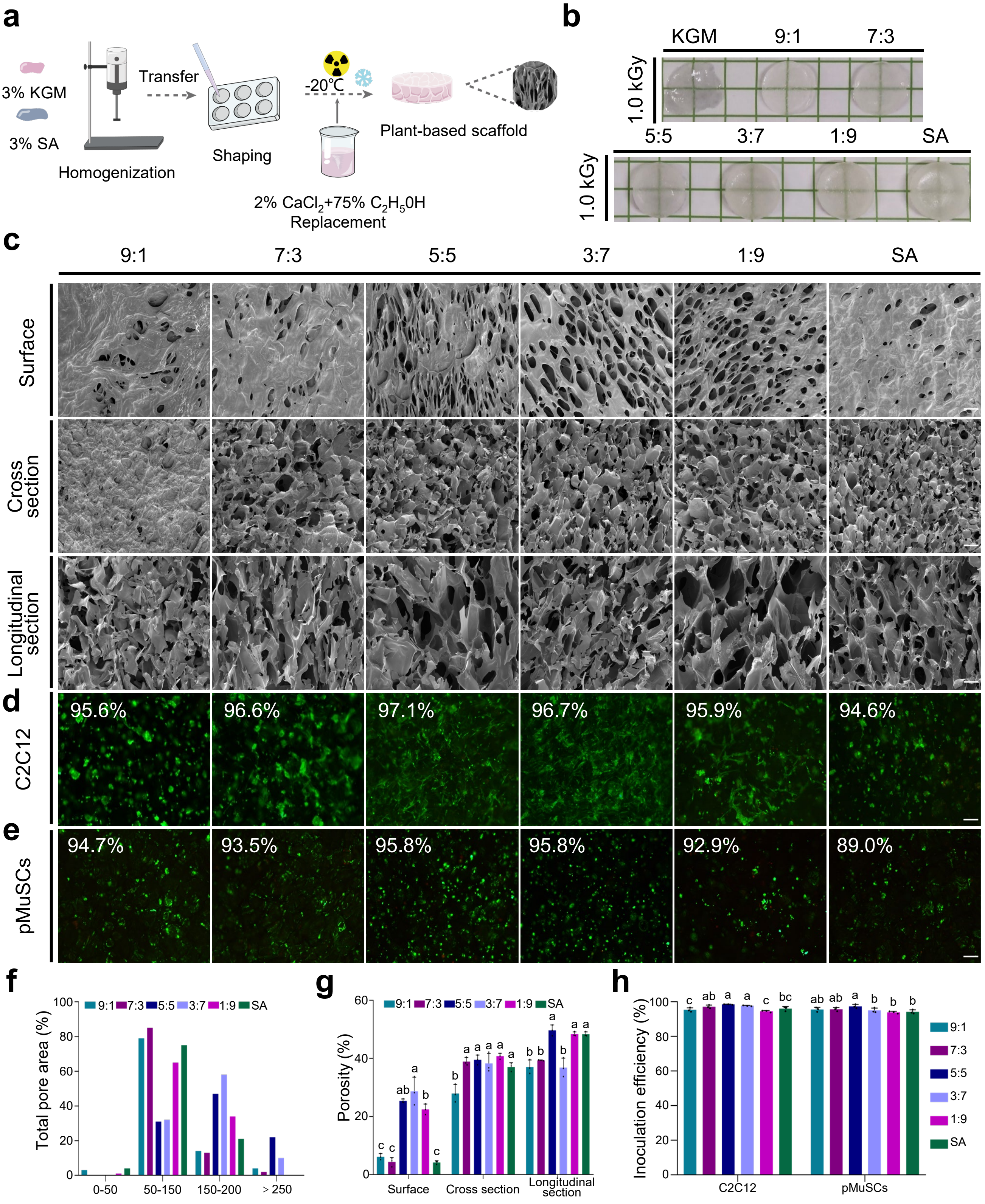

Supplementary Fig. 10: The optimization of plant-based 3D scaffolds composition (related to Fig. 5). **a** Schematic diagram of the preparation of the Ca^{2+}-KGM-SA scaffold. **b** The appearance of Ca^{2+}-KGM-SA scaffolds with different ratios. **c** Representative SEM images of the structure of the Ca^{2+}-KGM-SA scaffold surface (top), cross section (middle), and longitudinal section (bottom). Scale bar, 200 μm (top and middle), 100 μm (bottom). **d-e**. Representative images of Calcein-AM (green) and PI (red) of C2C12 and pMuSCs inoculated and cultured onto the Ca^{2+}-KGM-SA scaffold for 24 h. The percentage of live cells in the upper left corner was the result of the analysis of Calcein-AM and PI by flow cytometry. Scale bar, 100 μm. **f** Total pore size distribution of Ca^{2+}-KGM-SA scaffolds. **g** The porosity of the surface, cross-section and longitudinal section of Ca^{2+}-KGM-SA scaffolds. **h** Inoculation efficiency of C2C12 and pMuSCs on Ca^{2+}-KGM-SA scaffolds. For **a** and **b**, KGM: konjac glucomannan; SA: sodium alginate. For **g** and **h**, data was analyzed using a one-way ANOVA, followed by Duncan's new multiple range test. Data are presented as means ± SD (n = 3; a-c represent significant differences at $p < 0.05$). Exact P values are listed in Source Data Fig S10.

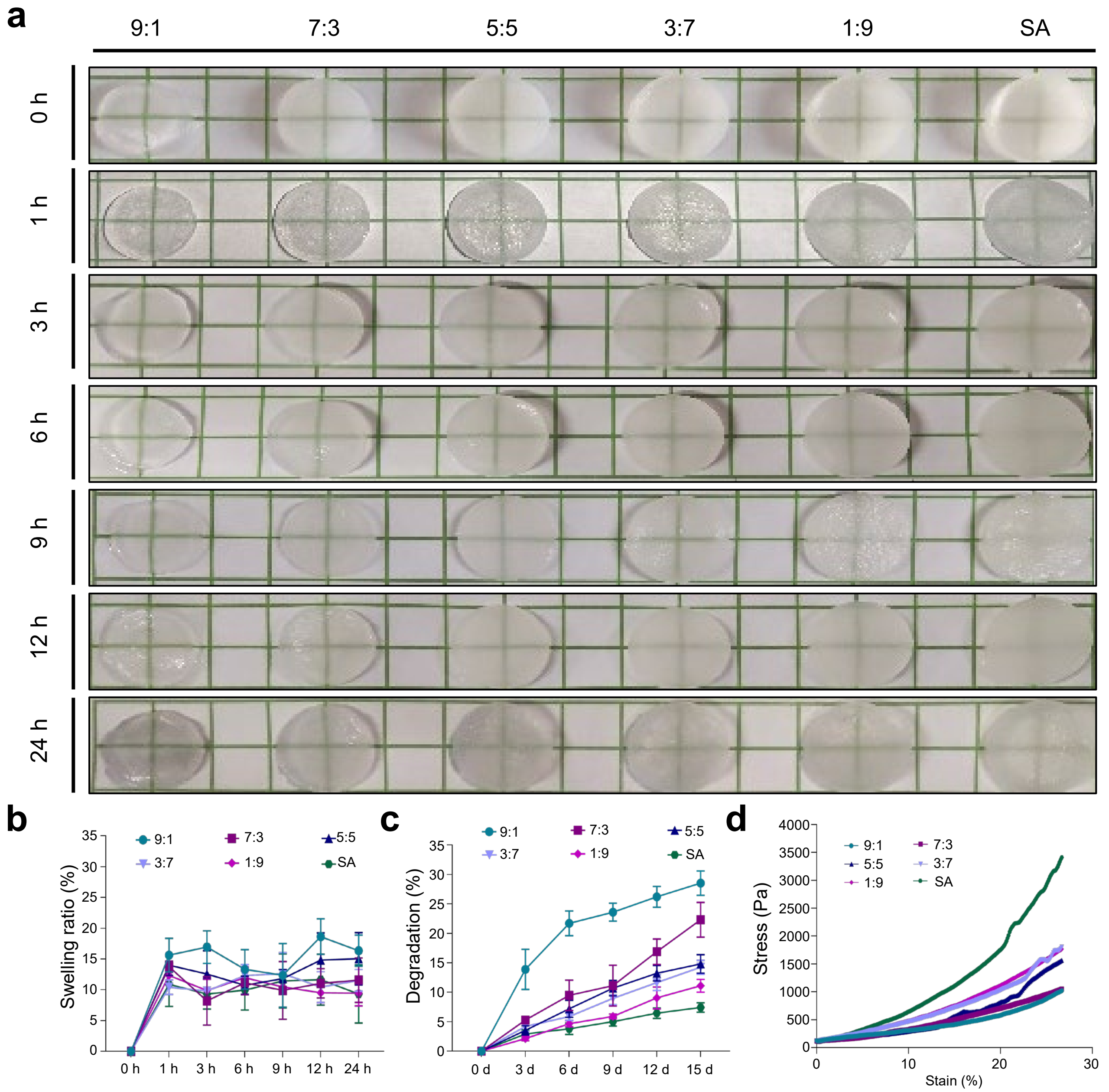

Supplementary Fig. 11: Physicochemical properties and mechanical properties analysis of plant-based 3D scaffolds (related to Fig. 5). a Images of the swelling behavior of Ca^{2+}-KGM-SA scaffolds. **b-d** Determination of the swelling ratio (**b**), degradation ratio (**c**), and compression stress-strain curves (**d**) of Ca^{2+}-KGM-SA scaffolds. Data are presented as means ± SD. For **a-d**, SA: sodium alginate. Data are listed in Source Data Fig S11.

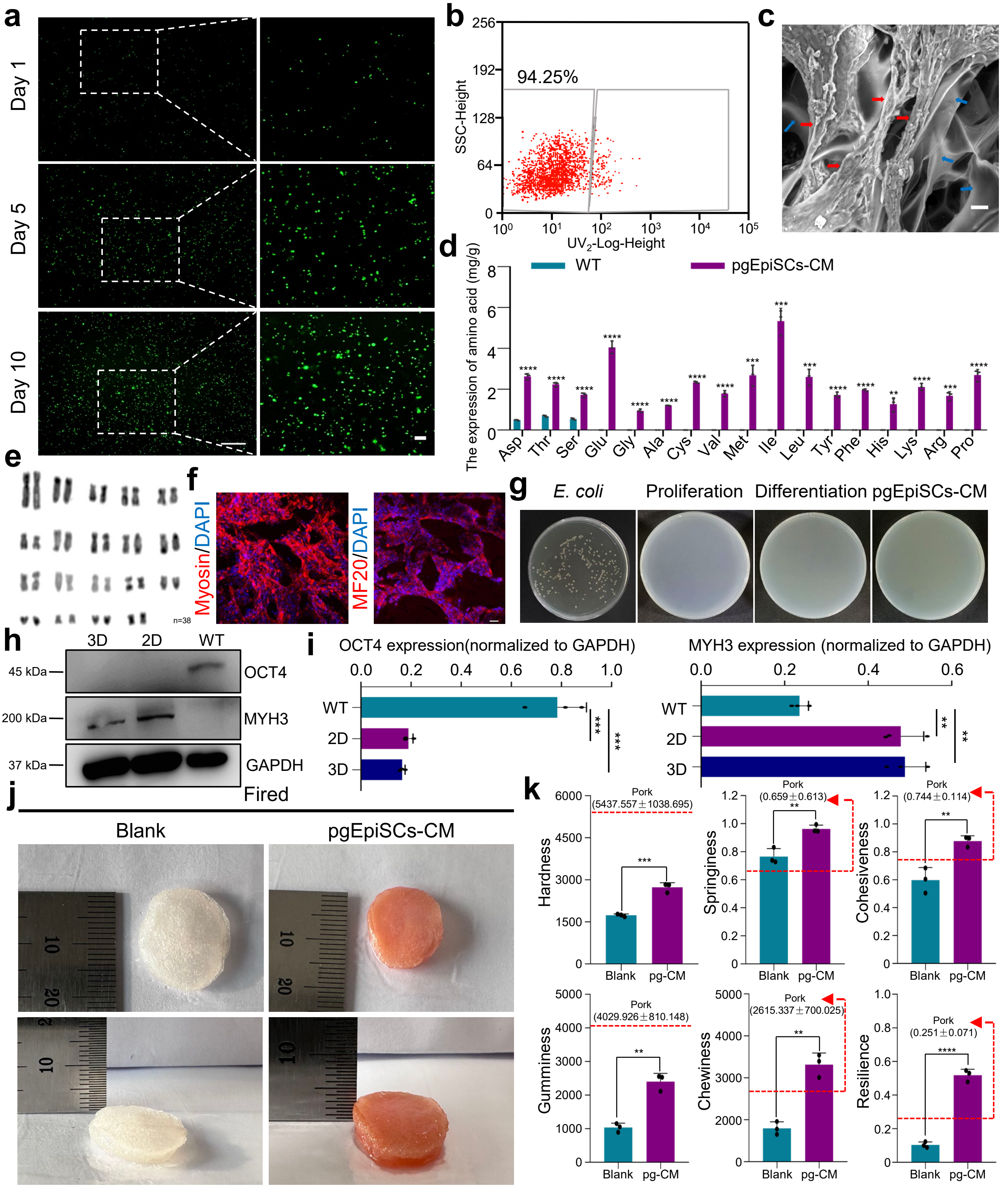

Supplementary Fig. 12: Proliferation capacity, stability, cleanliness and cooking characteristics of plant-based Ca^{2+}-KGM_5-SA_5 3D scaffolds (related to Fig. 5). a Proliferation of differentiated cells on 3D scaffolds was tracked with a reporter cell line (pgEpiSCs-NLS-GFP) in which the nuclei were marked with GFP. Scale bar, 500 μm (left) and 100 μm (right). **b** Flow cytometric assay for the survival of pgEpiSCs-MCs on 3D scaffolds for 24 h. **c** The interaction of pgEpiSCs-myoblast inoculated for 24 h with scaffolds was observed using SEM. Scale bar, 20 μm. Cells are indicated with red arrows, and the scaffold outline without cells attached is marked by blue arrows. **d** The amino acid contents of cultured meat tissue from pgEpiSCs were analyzed by an amino acid analyzer. Asp: Aspartate; Thr: Threonine; Ser: Serine; Glu: Glutamic Acid; Gly: Glycine; Ala: Alanine; Cys: Cysteine; Val: Valine; Met: Methionine; Ile: Isoleucine; Leu: Leucine; Tyr: Tyrosine; Phe: Phenylalanine; His: Histidine; Lys: Lysine; Arg: Argnine; Pro: Proline. **e** Karyotype analysis of pgEpiSCs-MCs after cultured on the 3D scaffolds, which maintain normal chromosome numbers (n=38). **f** Immunofluoresence staining of myotube differentiation on plant-based 3D edible scaffolds. MF20 or Myosin (red), DAPI (blue). Scale bar, 50 μm. **g** Microbiological detection of pgEpiSCs-derived CM. **h** Western blot analysis of OCT4, MYH3 and GAPDH expression in myogenic differentiation on 3D edible scaffolds. **i** Quantification of OCT4 and MYH3 expression normalized to GAPDH from western blot gel. WT: undifferentiated pgEpiSCs; 2D: myogenic differentiation of 2D; 3D: pgEpiSCs-MCs differentiation on 3D scaffolds. **j** Appearance of fried pgEpiSCs-derived CM. **k** Textural properties of fried pgEpiSC-derived CM. The red line indicates the value of the fried pork. Blank: empty scaffolds without cell inoculation; pg-CM: pgEpiSCs-derived CM. For **d**, **i** and **k**, Data are presented as mean ± SD, n = 3. ** $p < 0.01$, *** $p < 0.001$, **** $p < 0.0001$. Represent significant using two-tailed student's t test and similar results were obtained in three independent experiments. Exact *P* values are listed in Source Data Fig S12.

Supplementary information, Table 1, Key Resources Table.

Antibodies			
**REAGENT or RESOURCE**	**SOURCE**	**IDENTIFIER**	**DILUTION RATIO**
Mouse monoclonal anti-Oct-3/4	Santa Cruz Biotechnology	Cat# sc-5279	1:500
Mouse monoclonal anti-Sox2	Santa Cruz Biotechnology	Cat# sc-365823	1:300
Rabbit polyclonal anti-human Nanog	PeproTech	Cat# 500-P236	1:300
Rabbit polyclonal anti-neuron specific beta III Tubulin	Abcam	Cat# ab18207	1:250
Rabbit polyclonal anti-alpha smooth muscle Actin	Abcam	Cat# ab5694	1:250
Rabbit monoclonal anti-Vimentin	Abcam	Cat# ab92547	1:250
Goat polyclonal anti-Brachyury	Santa Cruz Biotechnology	Cat# sc17743	1:50
Rabbit monoclonal anti-human-γH2A.X	Cell Signaling Technology	Cat# 9718	1:1000
Mouse monoclonal anti-PAX7	DSHB	Cat# PAX7-S	1:100
Rabbit polyclonal anti-MYOD1	Proteintech	Cat# 18943-1-AP	1:200
Mouse monoclonal anti-Skeletal Myosin (Fast)	Sigma-Aldrich	Cat# M4276	1:300
Mouse monoclonal anti-Myosin heavy chain (MyHc)	DSHB	Cat# MF20-S	1:200
Mouse monoclonal anti-MYH3	Santa Cruz Biotechnology	Cat# sc-376157	1:1000
Rabbit monoclonal anti-GAPDH (D16H11)	Cell Signaling Technology	Cat# 5174	1:5000
Actin-Tracker Red-594	Beyotime	Cat# C2205S	1:100
DAPI	Roche Life Science	Cat# 10236276001	1:10000
Donkey anti-Rabbit IgG (H+L) highly Cross-Adsorbed Secondary Antibody, Alexa Fluor 594	Thermo Fisher Scientific	Cat# A-21207	1:1000
Donkey anti-Mouse IgG (H+L) highly Cross-Adsorbed Secondary Antibody, Alexa Fluor 594	Thermo Fisher Scientific	Cat# A-21203	1:1000
Donkey anti-Mouse IgG (H+L) highly Cross-Adsorbed Secondary Antibody, Alexa Fluor 488	Thermo Fisher Scientific	Cat# A-21202	1:1000
Horseradish peroxidase (HRP)-conjugated anti-rabbit IgG	Beyotime	Cat# A0208	1:10000
Horseradish peroxidase (HRP)-conjugated anti-mouse IgG	Cell Signaling Technology	Cat# 7076	1:10000
APC-conjugated anti-pig CD31	BIO-RAD	Cat# MCA1746APC	1:30
Alexa Fluor 647 anti-pig CD45	BIO-RAD	Cat# MCA1222A647	1:30
PEconjugated anti-human CD56	BioLegend	Cat# 304606	1:50
StarGreen safe Nucleic Acid Dye 10000×	GenStar	Cat# ZE111-10	1:10000

Chemicals, Peptides and Recombinant Protein		
**REAGENT or RESOURCE**	**SOURCE**	**IDENTIFIER**
CHIR99021	Selleckchem	Cat# S1263
IWR-1-endo	Selleckchem	Cat# S7086
Y-27632	Selleckchem	Cat# S1049
WH-4-023	Selleckchem	Cat# S7565
Recombinant Human LIF	PeproTech	Cat# 300-05
Human/Murine/Rat Activin A	PeproTech	Cat# 120-14E
Recombinant Human FGF-2	PeproTech	Cat# 100-18B
Recombinant Human IGF-1	PeproTech	Cat# 100-11
Recombinant Human HGF	PeproTech	Cat# 100-39H
SB431542	Selleckchem	Cat# S1067
LDN193189	Stemgent	Cat# 04-0074
Bovine Serum Albumin (BSA)	Sigma-Aldrich	Cat# A1470
Ascorbic Acid (Vc)	Sigma-Aldrich	Cat# A4544
KnockOut Serum Replacement	Thermo Fisher Scientific	Cat# A3181502
Neurobasal™ Medium	Thermo Fisher Scientific	Cat# 21103-049
DMEM/F12, GlutaMAX™ supplement	Thermo Fisher Scientific	Cat# 10565-018
N-2 Supplement (100 ×)	Thermo Fisher Scientific	Cat# 17502-048
B-27 ™ Supplement (50 ×), minus vitamin A	Thermo Fisher Scientific	Cat# 12587-010
Insulin–transferrin–selenium (ITS)	Thermo Fisher Scientific	Cat# 41400-045
GlutaMAX™ Supplement	Thermo Fisher Scientific	Cat# 35050-061

2-Mercaptoethanol	Thermo Fisher Scientific	Cat# 21985-023
MEM Non-Essential Amino Acids Solution (100 ×)	Thermo Fisher Scientific	Cat# 1140-050
Penicillin-Streptomycin (10,000 U/mL)	Thermo Fisher Scientific	Cat# 15140-122
Gelatin (0.1 % in water)	Stem Cell Technologies	Cat# 07903
Trypsin-EDTA (0.05%), phenol red	Gibco	Cat# 25300120
DMEM, high glucose, no glutamine	Gibco	Cat# 11960-044
Fetal bovine serum (FBS)	Gibco	Cat# 16000-044
Horse serum	Gibco	Cat# 26050088
Accutase cell dissociation reagent	Gibco	Cat# A11105-01
TrypLE™ Express	Gibco	Cat# 12605010
Dispase II	Coolaber	Cat# CD4691
Collagenase II	Coolaber	Cat# CC3791G
Calcium chloride	Sigma-Aldrich	Cat# C5670
RIPA	Cell Signaling Technology	Cat# 9806
Protease inhibitor cocktail	Beyotime	Cat# P1050
SuperSignal™ West Dura Extended Duration Substrate	Thermo Fisher Scientific	Cat# 34075
H_2O_2	Sigma-Aldrich	Cat# 316989
Colcemid Solution	Gibco	Cat# 15210-040
Mitomycin C	Selleckchem	Cat# S8146
Red Blood Cell Lysis Buffer	Solarbio	Cat# R1010
Dulbecco's phosphate-buffered saline (DPBS)	Gibco	Cat# C14190500CP
Hanks' Balanced Salt Solution (with Ca^{2+} & Mg^{2+})	Beyotime	Cat# C0219
Sodium alginate	Sinopharm Chemical Reagent Co.,Ltd.	Cat# 9005-38-3
Konjac glucomnnan	Hubei konson Konjac Technology Co.	Cat# KJ30

**Primer**		
OCT4:<br>F: CAAACTGAGGTGCCTGCCCTTC<br>R: ATTGAACTTCACCTTCCCTCCA	This paper	N/A
SOX2:<br>F: CATCAACGGTACACTGCCTCTC<br>R: ACTCTCCTCCCATTTCCCTCTT	This paper	N/A
NANOG:<br>F: CATCTGCTGAGACCCTCGAC<br>R: GGGCTTGTGGAAGAATCAGG	This paper	N/A
T:<br>F: GCCAGATCATGCTGAACTCCTTA<br>R: ATAAGCCGTCACCGCTATGAAC	This paper	N/A
MSGN1:<br>F: AACTACCTGCCACCGGTCTA<br>R: TCCGCTGTTGAGAAGGTCTG	This paper	N/A
PDGFRα:<br>F: GACAGTGCTGGAAGTGGTCA<br>R: TCCGGGTCTGGCACATAGAT	This paper	N/A
PAX6:<br>F: TGTCCAACGGATGTGTGAGT<br>R: TCTGTCTCGGATTTCCCAA	This paper	N/A
EOMES:<br>F: ACTCCCATGGACCTCCAGAA<br>R: TCGCTTACAAGCACTGGTGT	This paper	N/A
BMP4:<br>F: TTCATTTTAGGAGCCATTCTGTAGT<br>R: TCCTAGCAGGACTTGGCATAAT	This paper	N/A
PAX7:<br>F: GTGCCCTCAGTGAGTTCGATTA<br>R: TTCCCTTTGTCGCCCAAGAT	This paper	N/A
MYOD:<br>F: CGCTTGAGCAAAGTCAACGA<br>R: GCTATAATCCATCATGCCGTCG	This paper	N/A
MYF5:<br>F: ACGGCATGCCTGAATGCAAC<br>R: TGCTGATCCGATCCACTATGC	This paper	N/A

MYOG:<br>F: CCAGGGGATCATCTGCTCACA<br>R: TGGGCATGGTTTCATCTGGG	This paper	N/A
MYMK:<br>F: CTTCCTCCCCACGGTCAG<br>R: TACTCCAGGATGTCAAGGCG	This paper	N/A
MYH1:<br>F: TCTTTGACTGGGCTGCCATC<br>R: TTCACTGTCAAAGTCGCCCC	This paper	N/A
MYH2:<br>F: GGGCTCAAACTGGTGAAGC<br>R: AGATGCGGATGCCCTCCA	This paper	N/A
MYH3:<br>F: GCCGACGCTGACAGCGGAAA<br>R: AGATGCGGATGCCCTCCA	This paper	N/A
MEF2A:<br>F: CACGGGGTGACTTCCATTCT<br>R: CAGGGTGCTCTGTTTGCCAT	This paper	N/A
MEF2C:<br>F: GACAACAAAGCCCTCAGCAG<br>R: CTGCATTCGTTCCTGCACAT	This paper	N/A
ACTN2:<br>F: CAGCAAAGGCGTGAAACTGG<br>R: TGGCAGAGGTTTCTTCGACT	This paper	N/A
MYL3:<br>F: GCCCAAGAAGGATGATGCCA<br>R: TCACACTTGGGTGTACGGTC	This paper	N/A
MYL4:<br>F: CACATCTCCCGCAACAAGGA<br>R: TCTTCCTGCTCCACCCTG	This paper	N/A
MYH8:<br>F: TCTGGCATCCAGAGGCAGAAT<br>R: GTTAGCGTTGCTCCACGTTC	This paper	N/A
MYH11:<br>F: GAGCGCCACATCTCAACTCT<br>R: CTCCTCGGCCAACAACTGAT	This paper	N/A
CD31:<br>F: CACCGAGGTCTGGGAACAAA<br>R: GGGAGCCTTCCGTTCTAGAATATC	This paper	N/A
CDH5:<br>F: AAGAACATCGCCCCGTGTCAT<br>R: CACTGAGCCGATCCAAGGTT	This paper	N/A
CD90:<br>F: GGCATCGCTCTCTTGCTAAC<br>R: GGACCTTGATGTCGTACTTGC	This paper	N/A
CD105:<br>F: ATGCTGTCGTAGCAACCCAA<br>R: GCCGGACCTCTTCTGTTCTC	This paper	N/A
COL11A1:<br>F: ACTGTCATCAGTCAGCAGCC<br>R: TAGCCTTTTCTGGACGCACA	This paper	N/A
FBN1:<br>F: ATGAATGTCTGGAGCCACCC<br>R: TCCAGAGCGGGTATCAACAC	This paper	N/A
COL6A3:<br>F: AGCATCGGACCAAAGGGAAT<br>R: TGGAAACCTTGAGTGCCGTT	This paper	N/A
COL5A2:<br>F: AAACTGGGCAGAAGCAAGAC<br>R: ATTTCTTCACCATATCCTTCATCCT	This paper	N/A
COL3A1:<br>F: CTAGCCGAGCTTCCCAGAAC<br>R: TCCCCAGTGTGTTTAGTGCAA	This paper	N/A
LAMA4:<br>F: TCCTGCTACCCAGAAATGCG<br>R: CTCTCCTGTTGTGTTCCGCT	This paper	N/A
ELN:<br>F: GAGCCTTTGGAGGTGTGTCC<br>R: CGCCACCTGGGAAAATAGGA	This paper	N/A
PKM:<br>F: CCAGAAACCAAGCAGCAACA<br>R: AAGTCACTCGCTGTCCTTCA	This paper	N/A
PGK1:<br>F: GAATGGACCTGTGGGCGTAT<br>R: CAGTGTCTCCACCACCTATGA	This paper	N/A

PGAM1:<br>F: GCAACATCAGTAAGGATCGCA<br>R: GCTCCATGATCGCCTCTTCA	This paper	N/A
LDHA:<br>F: AAGTGCACTCCCGATTCCTTT<br>R: AGGAAAAGGCTGCCATGTTG	This paper	N/A
PDK4:<br>F: ACCAGGAAAACTGGCCTTCT<br>R: CCGTAACCAAAACCAGCCAAAG	This paper	N/A
PDP1:<br>F: CCCAGACGAATTGGAACCCC<br>R: GGATGGGGCGTGTATCTCAG	This paper	N/A
PDHA1:<br>F: TTAGGTCTTAGCGTCCTCCC<br>R: GCCGGTGAAGATCACATTTCTTA	This paper	N/A
HK1:<br>F: AGCTTCATCCACACTTCTCCA<br>R: GCACAGCTCGCTAGACAGAA	This paper	N/A
HK2:<br>F: CTCCGGATGGGACAGAACAC<br>R: TGAAGTTAGCCAGGCACTCG	This paper	N/A
SLC2A11:<br>F: AATGCCCCAACCTTGCACAT<br>R: TTCCTTCCCAGCTTGATGGC	This paper	N/A
SLC2A12:<br>F: AGTCTGCTGAAACCGAAGGG<br>R: GTAAACATGCCGCAACCTCTC	This paper	N/A
SLC2A13:<br>F: GCTCGGCATCGGTATTGCT<br>R: CCTCCCGTGATGAAGAGGGT	This paper	N/A
PGM1:<br>F: CGGAATTTCTTCACCAGGTACG<br>R: AATGCTTCCGTCCACAGGAT	This paper	N/A
GPI:<br>F: TTCGAGTTCTGGGATTGGGTG<br>R: TTCTTCTCCAGGGGTGTCGT	This paper	N/A
EF-1α:<br>F: AATGCGGTGGGATCGACAAA<br>R: CACGCTCACGTTCAGCCTTT	This paper	N/A

**Critical Commercial Assays**		
**REAGENT or RESOURCE**	**SOURCE**	**IDENTIFIER**
RNApre pure Cell / Bacteria Kit	TIANGEN	Cat# DP430
Hifair® III 1st Strand cDNA Synthesis SuperMix for qPCR (gDNA digester plus)	YEASEN	Cat# 11141ES60
2 × RealStar Green Power Mixture	GenStar	Cat# A311-05
Alkaline Phosphatase Detection Kit	Millipore	Cat# SCR004
Rapid Giemsa Staining kit	BBI Life Science	Cat# E6073141
Calcein / PI Cell Activity and Cytotoxicity Assay Kit	Beyotime	Cat# C2015M
Modified BCA Protein Assay Kit	Sangon Biotech	Cat# C503051
ATP Assay Kit	Beyotime	Cat# S0027
Glucose Assay Kit	Beyotime	Cat# S0201M
Lactic Acid assay kit	Nanjing Jiancheng institute of Biotechnology	Cat# A019-2-1
TIANSeq mRNA capture kit	TIANGEN	Cat# NR105
VAHTS Universal V6 RNA-seq Library Prep Kit for Illumina	VAZYME	Cat# NR604-02

**Configuration of culture medium**
(1) pgEpiSCs culture medium
Basic culture medium:227.5 mL DMEM/F12, 227.5 mL neurobasal, 2.5 mL N2 supplement, 5 mL B27 supplement, 0.5% GlutaMAX, 1% nonessential amino acids, 0.1 mM β-mercaptoethanol, 1% penicillin–streptomycin, 5% knockout serum replacement, and 50 μg/mL ascorbic acid.<br>3i/LAF medium: small molecules and cytokines were added to BM to the following final concentrations, 1 μM

CHIR99021, 2.5 μM IWR-1-endo, 1 μM WH-4-023, 10 ng/mL recombinant human LIF, 25 ng/mL recombinant human activin A, 10 ng/mL recombinant human FGF2.
(2) pMuSCs culture and differentiation medium
Culture medium: DMEM/F12 supplemented with 1% penicillin-streptomycin, 1% MEM Non-Essential Amino Acids Solution, 10% FBS, and 5 ng/mL FGF2.<br>Differentiation medium: DMEM supplemented with 1% penicillin-streptomycin and 2% Horse serum.
(3) Embryoid body differentiation medium
DMEM supplemented with 10 % FBS, 1 % penicillin–streptomycin, 1 % GlutaMAX.
(4) Adherent medium
ITS group (appropriate adjustments): DMEM/F12 supplemented with 1% ITS, 0.1 mM β-mercaptoethanol ,1% nonessential amino acids, 1% penicillin–streptomycin and 200 μM ascorbic acid.<br>YH BM group (appropriate adjustments): DMEM/F12 and neurobasal (1:1) supplemented with 0.5% N2 supplement, 1% B27 supplement, 1% nonessential amino acids, 0.1 mM β-mercaptoethanol, 1% penicillin–streptomycin, 15% knockout serum replacement, and 200 μM ascorbic acid.<br>YHBM-Neur: DMEM/F12 supplemented with 0.5% N2, 1% B27, 1% nonessential amino acids, 0.1 mM β-mercaptoethanol, 1% penicillin–streptomycin, 15% knockout serum replacement, and 200 μM ascorbic acid.<br>F12+N2 group: DMEM/F12 supplemented with 0.5% N2, 1% nonessential amino acids, 0.1 mM β-mercaptoethanol, 1% penicillin–streptomycin, 15% knockout serum replacement, and 200 μM ascorbic acid.<br>F12+B27 group: DMEM/F12 supplemented with 1% B27, 1% nonessential amino acids, 0.1 mM β-mercaptoethanol, 1% penicillin–streptomycin, 15% knockout serum replacement, and 200 μM ascorbic acid.
(5) Myogenic differentiation medium (MDM)
MDM basic medium (MDM BM): DMEM/F12 supplemented with 1% nonessential amino acids, 0.1 mM β-mercaptoethanol, 1% penicillin–streptomycin, 15% knockout serum replacement, 200 μM ascorbic acid.<br>MDM I (Optimization of MDM I): a) MDM BM supplemented with 1% B27, 3 μM CHIR99021 and 0.5 μM LDN193189; b) MDM BM supplemented with 1% B27, 3 μM CHIR99021 and 2 μM SB431542; c) MDM BM supplemented with 1% B27, 3 μM CHIR99021, 0.5 μM LDN193189 and 2 μM SB431542.<br>MDM II: MDM BM supplemented with 3 μM CHIR99021, 0.5 μM LDN193189, and 20 ng/ml FGF2.<br>MDM III: MDM BM supplemented with 10 ng/ml HGF, 10 ng/ml IGF-1, 20 ng/ml FGF2 and 0.5 μM LDN193189.<br>MDM IV: MDM BM supplemented with 10 ng/ml IGF-1.<br>MDM V: MDM BM supplemented with 10 ng/ml HGF and 10 ng/ml IGF-1.<br>N2 medium: DMEM/F12 supplemented with 15% KOSR, 1% N2, 1% penicillin–streptomycin, and 1% nonessential amino acids.<br>HS medium: DMEM/F12 supplemented with 15% KOSR, 2% HS, 1% penicillin–streptomycin, and 1% nonessential amino acids.

**Deposited Data**	
pgEpiSCs Low and High passages RNA-seq data	This articile
pgEpiSCs, pgEpiSCs-MPCs and pgEpiSCs-MCs RNA-seq data	This articile
Muscle progenitor cells (MPCs) differentiation RNA-seq data	This articile
PEFs	[1]
**Software and Algorithms**	
Trim garole	[2]
HISAT2	[3]
FeatureCounts	[4]
DEseq2 tool	[5]
R package FactoMineR	[6]
R package factoextra	[7]
R packages pheatmap	[8]
R packages EnhancedVolcano	[9]
R package ggtern	[10]
Metascape	[11]
ggplot2	[12]
ProteoWizard	[13]
XCMS	[14]
IP4M	[15]
mixOmics	[16]

Supplementary Table 2 Analyzing the nutritional composition of pgEpiSCs-CM.

Items	Scaffold (without cells)	pgEpiSCs-CM
H_2O	95.82 %	95.74 %
Total Protein	No	3.92 %
Total Fat	No	No
Zinc (Zn)	156.89 μg/Kg	999.67 μg/Kg
Calcium (Ca)	14539.67 μg/Kg	1708.78 μg/Kg
Iron (Fe)	16.17 μg/Kg	5.02 μg/Kg
Magnesium (Mg)	9.76 μg/Kg	57.564 μg/Kg
Potassium (K)	0.47 μg/Kg	271.26 μg/Kg

Determination of the protein, fat, moisture, and trace elements in pgEpiSCs-CM samples

The protein content of pgEpiSCs-CM was determined following the protocol outlined in GB5009.5-2016. Specifically, 2 g of the sample was accurately weighed and placed into a digestion tube for digestion in a furnace. The temperature was raised to 420°C and maintained for one hour until the liquid turned green and transparent before being removed, cooled, distilled, and titrated using an automatic Kjeldahl nitrogen analyzer (Kjeltec 8400, FOSS Ltd., Denmark).

The fat content in pgEpiSCs-CM was determined following the protocol outlined in GB5009.6-2016. Specifically, 5 g of the sample was weighed into an evaporating dish and mixed with approximately 20 g of quartz sand. The mixture was then evaporated in a boiling water bath and subsequently dried at 100 °C ± 5°C for 30 min using an electric blast drying oven. To extract the processed samples, a Soxhlet extractor was employed, refluxing with anhydrous ether for eight cycles. The completion of the extraction was indicated by no oil spots observed when picking up one drop of the extract with a frosted glass rod.

Detect the moisture content in pgEpiSCs-CM by GB5009.3-2016. Accurately weigh 2 g of the sample, flat in the dry, to a constant weight of the flat weighing bottle with a thickness of not more than 5 mm and no more than 10 mm of loose test material. The weighing bottle after weighing the test material was placed in an oven that had been raised to 100~105°C, dried for 5 h, removed, transferred to a desiccator, cooled at room temperature for 30 min, precision weighed. The difference between two consecutive weighings did not exceed 5 mg until.

Multiple elements in cells were determined according to GB 5009.268-2016. Basically, 1.0 mL of samples were accurately measured and placed in a cleaned polytetrafluoroethylene (PTFE) digestion tank. Each sample was sealed with 0.5 mL of nitric acid (KERMEL, CAS:7697-37-2), and the microwave digestion procedure was performed. After digestion, the samples were transferred to clean PET plastic bottles, diluted to 2.0 g with ultra-pure water, and treated in the same manner as the blank controls. The elements of the sample were determined by ICP-MS (ICAP-QC, Thermo Fisher Scientific, Waltham, Massachusetts, USA).

Supplementary references

1. Xu, J. *et al.* Generation of pig induced pluripotent stem cells using an extended pluripotent stem cell culture system. *Stem Cell Res. Ther.* **10**, 193 (2019).
2. Martin, M. Cutadapt removes adapter sequences from high-throughput sequencing reads. *EMBnet J.* **17**, 10-12 (2011).
3. Pertea, M., Kim, D., Pertea, G.M., Leek, J.T. & Salzberg, S.L. Transcript-level expression analysis of RNA-seq experiments with HISAT, StringTie and Ballgown. *Nat. Protoc.* **11**, 1650-1667 (2016).
4. Liao, Y., Smyth, G.K. & Shi, W. featureCounts: an efficient general purpose program for assigning sequence reads to genomic features. *Bioinformatics* **30**, 923-930 (2014).
5. Love, M.I., Huber, W. & Anders, S. Moderated estimation of fold change and dispersion for RNA-seq data with DESeq2. *Genome. Biol.* **15**, 550 (2014).
6. Lê, S., Josse, J. & Husson, F. FactoMineR: An R Package for Multivariate Analysis. *J. Stat. Softw.* **25**, 1-18 (2008).
7. Kassambara, A., and Mundt, F. Package ‘factoextra’. Extract and visualize the results of multivariate data analyses. 76 (2017).
8. Kolde, R. pheatmap: Pretty Heatmaps. R package version 1.0. 12. CRAN R-project org/package= pheatmap. (2019).
9. Blighe, K., Rana, S., and Lewis, M.. EnhancedVolcano: Publication-ready volcano plots with enhanced colouring and labeling. R package version 1 (2019).
10. Hamilton, N.E. & Ferry, M.J.J.o.s.s. ggtern : Ternary Diagrams Using ggplot2. *J. Stat. Softw.* **87**, 1-17 (2018).
11. Zhou, Y. *et al.* Metascape provides a biologist-oriented resource for the analysis of systems-level datasets. *Nat. Commun.* **10**, 1523 (2019).
12. Hadley, W. Data analysis. In ggplot2 (Springer), pp. 189-201 (2016).
13. Darren, K., Matt, C., Robert, B., David, A. & Parag, M.J.B.-O.-. ProteoWizard: open source software for rapid proteomics tools development. *Bioinformatics* **24**, 2534-2536 (2008).
14. Want, E.J, *et al.* XCMS: Processing Mass Spectrometry Data for Metabolite Profiling Using Nonlinear Peak Alignment, Matching, and Identification. *Anal. Chem.* **78**, 779-787 (2006).
15. Liang, D. *et al.* IP4M: an integrated platform for mass spectrometry-based metabolomics data mining. *BMC Bioinformatics* **21**, 444 (2020).
16. Rohart, F., Gautier, B., Singh, A. & Cao, K.J.P.C.B. mixOmics: An R package for 'omics feature selection and multiple data integration. *PLoS Comput. Biol.* **13**, e1005752 (2017).
