# Supplementary material for: Generation of three-dimensional meat-like tissue from stable pig epiblast stem cells
Source: Nat Commun. 2023 Dec 9;14:8163. doi: 10.1038/s41467-023-44001-8 (PMC10710416; doi:10.1038/s41467-023-44001-8)
Supplement: Supplementary file 3 — Reporting Summary [file 41467_2023_44001_MOESM3_ESM.pdf]

nature portfolio

Corresponding author(s): Jianyong Han, Xianchao Feng, Aijin Ma.

Last updated by author(s): Oct 27, 2023

Reporting Summary

Nature Portfolio wishes to improve the reproducibility of the work that we publish. This form provides structure for consistency and transparency in reporting. For further information on Nature Portfolio policies, see our Editorial Policies and the Editorial Policy Checklist.

Statistics

For all statistical analyses, confirm that the following items are present in the figure legend, table legend, main text, or Methods section.

n/a	Confirmed	
☐	☒	The exact sample size (*n*) for each experimental group/condition, given as a discrete number and unit of measurement
☐	☒	A statement on whether measurements were taken from distinct samples or whether the same sample was measured repeatedly
☐	☒	The statistical test(s) used AND whether they are one- or two-sided<br>*Only common tests should be described solely by name; describe more complex techniques in the Methods section.*
☐	☒	A description of all covariates tested
☐	☒	A description of any assumptions or corrections, such as tests of normality and adjustment for multiple comparisons
☐	☒	A full description of the statistical parameters including central tendency (e.g. means) or other basic estimates (e.g. regression coefficient) AND variation (e.g. standard deviation) or associated estimates of uncertainty (e.g. confidence intervals)
☐	☒	For null hypothesis testing, the test statistic (e.g. *F*, *t*, *r*) with confidence intervals, effect sizes, degrees of freedom and *P* value noted<br>*Give P values as exact values whenever suitable.*
☒	☐	For Bayesian analysis, information on the choice of priors and Markov chain Monte Carlo settings
☒	☐	For hierarchical and complex designs, identification of the appropriate level for tests and full reporting of outcomes
☐	☒	Estimates of effect sizes (e.g. Cohen's *d*, Pearson's *r*), indicating how they were calculated

Our web collection on statistics for biologists contains articles on many of the points above.

Software and code

Policy information about availability of computer code

Data collection	The data of RT-PCR was collected by Archimed X6 (ROCGENE, China);<br>The data of morphology of cells on scaffolds, fluorescence and fluorescence were collected by inverted microscopy (OLYMPUS, CKX41), scanning electron microscopy (HITACHI, TM-4000 plus).<br>The data for ATP, glucose and lactic acid measuring was collected by Multimode reader (Tecan, spark).<br>The data of cell viability and differentiation efficiency of pgEpiSCs were collected by BD FACSVerse (BD Biosciences, USA).<br>The Images of pgEpiSCs-MCs on 3D edible scaffolds were taken using a laser scanning confocal microscope (Lecai STELLARIS5, Germany).<br>Texture profile analysis (TPA) of scaffolds inoculated pgEpiSCs-MCs was measured by a texture analyzer (TA. XT Plus, Stable Micro systems Ltd, UK).<br>The determination of individual amino acids by using a fully automated amino acid analyzer (LA8080, Hi-tachi, Tokyo, Japan) for comparison with the standard (013-08391. Wako, Tokyo, Japan).<br>For transcriptome sequencing: the captured RNA as the initial sample for library construction with the VAHTS Universal V6 RNA-seq Library Prep Kit for Illumina (VAZYME, Cat# NR604-02).<br>All LC/MS acquired raw data were converted to mzXML using ProteoWizard (V3.0.22248).
Data analysis	**Image data:<br>ImageJ (Fiji, version 1.8.0, National Institute of Health, USA)<br>GraphPad Prism 9 (version 9.5.1, GraphPad Software, USA)<br>FlowJo (V10)<br>BioSys Manual 4.1.2 (Biochrom Ltd)<br>** RNA-seq data:

trim garole (v-0.6.6)
HISAT2 (v-2.1.0)
FeatureCounts (v-2.0.1)
DESeq2 (v-1.30.1)
FactoMineR (v-2.4) factoextra (v-1.0.7)
pheatmap (v-1.0.12)
EnhancedVolcano (v-1.14.0)
ggtern (v-3.3.5)
Metascape (http://metascape.org)
** Metabolomics data:
ProteoWizard (v-3.0.22248)
XCMS (v-3.10.2)
mzCloud (https://www.mzcloud.org/)
Compound Discoverer (v-3.0)
IP4M (v-2.0)
mixOmics (v-6.14.1)
ggplot2 (v-3.3.6)
R (v-4.0.5)

For manuscripts utilizing custom algorithms or software that are central to the research but not yet described in published literature, software must be made available to editors and reviewers. We strongly encourage code deposition in a community repository (e.g. GitHub). See the Nature Portfolio guidelines for submitting code & software for further information.

Data

Policy information about availability of data

All manuscripts must include a data availability statement. This statement should provide the following information, where applicable:

- Accession codes, unique identifiers, or web links for publicly available datasets
- A description of any restrictions on data availability
- For clinical datasets or third party data, please ensure that the statement adheres to our policy

Data are available in the main text, supplementary materials, Gene Expression Omnibus (GSE223433) and Open Archive for Miscellaneous Data (OMIX005128).

Research involving human participants, their data, or biological material

Policy information about studies with human participants or human data. See also policy information about sex, gender (identity/presentation), and sexual orientation and race, ethnicity and racism.

Reporting on sex and gender	N/A
Reporting on race, ethnicity, or other socially relevant groupings	N/A
Population characteristics	N/A
Recruitment	N/A
Ethics oversight	N/A

Note that full information on the approval of the study protocol must also be provided in the manuscript.

Field-specific reporting

Please select the one below that is the best fit for your research. If you are not sure, read the appropriate sections before making your selection.

[x] Life sciences [ ] Behavioural & social sciences [ ] Ecological, evolutionary & environmental sciences

For a reference copy of the document with all sections, see nature.com/documents/nr-reporting-summary-flat.pdf

Life sciences study design

All studies must disclose on these points even when the disclosure is negative.

Sample size	No sample-size calculation was performed. The sample size for each analysis was determined to be the minimum sample size required to make valid inferences, such as differences or similarity between different groups, which were deemed sufficient for robust P-value calculations and enabled us to conclude that the sample size in our experiments was enough.
Data exclusions	No data were excluded from the analyses.
Replication	The experiments were replicated or performed independently based on the numbers mentioned in the manuscript (such as methods section

and figure legends).

Randomization	In this study, there were no clinical populations or patients, therefore randomization techniques were not applicable. The same well plate the experiment was conducted in by dividing into treatments at random, which collects images of samples and experimental data.
Blinding	Blinding is not applicable to the current work since clinical populations or patients were not involved. The data (such as qPCR, fluorescence area, western blot band intensity, etc.) obtained were based on objective measurements, hence blinding has no impact on these data values.

Reporting for specific materials, systems and methods

We require information from authors about some types of materials, experimental systems and methods used in many studies. Here, indicate whether each material, system or method listed is relevant to your study. If you are not sure if a list item applies to your research, read the appropriate section before selecting a response.

Materials & experimental systems

n/a	Involved in the study
☐	☒ Antibodies
☐	☒ Eukaryotic cell lines
☒	☐ Palaeontology and archaeology
☐	☒ Animals and other organisms
☒	☐ Clinical data
☒	☐ Dual use research of concern
☒	☐ Plants

Methods

n/a	Involved in the study
☒	☐ ChIP-seq
☐	☒ Flow cytometry
☒	☐ MRI-based neuroimaging

Antibodies

Antibodies used

Conjugated first antibodies for FACS:
APC-conjugated anti-pig CD31 (BIO-RAD, Cat# MCA1746APC, dilution 1/30)
Alexa Fluor 647 anti-pig CD45 (BIO-RAD, Cat# MCA1222A647, dilution 1/30)
PE-conjugated anti-human CD56 (BioLegend, Cat# 304606, dilution 1/50)

The antibodies for immunofluorescence staining:
Mouse monoclonal anti-Oct-3/4 (Santa Cruz Biotechnology, Cat# sc-5279, dilution 1/500)
Mouse monoclonal anti-Sox2 (Santa Cruz Biotechnology, Cat# sc-365823, dilution 1/300)
Rabbit polyclonal anti-human Nanog (PeproTech, Cat# 500-P236, dilution 1/300)
Rabbit polyclonal anti-neuron specific beta III Tubulin (Abcam, Cat# ab18207, dilution 1/250)
Rabbit polyclonal anti-alpha smooth muscle Actin (Abcam, Cat# ab5694, dilution 1/250)
Rabbit monoclonal anti-Vimentin (Abcam, Cat# ab92547, dilution 1/250)
Goat polyclonal anti-Brachyury (Santa Cruz Biotechnology, Cat# sc17743, dilution 1/50)
Rabbit monoclonal anti-human-γH2A.X (Cell Signaling Technology, Cat# 9718, dilution 1/1000)
Mouse monoclonal anti-PAX7 (DSHB, Cat# PAX7-S, dilution 1/100)
Rabbit polyclonal anti-MYOD1 (Proteintech, Cat# 18943-1-AP, dilution 1/200)
Mouse monoclonal anti-Skeletal Myosin (Fast) (Sigma-Aldrich, Cat# M4276, dilution 1/300)
Mouse monoclonal anti-Myosin heavy chain (MyHc) (DSHB, Cat# MF20-S, dilution 1/200)
Actin-Tracker Red-594 (Beyotime, Cat# C2205S, dilution 1/100)
Donkey anti-Rabbit IgG (H+L) highly Cross-Adsorbed Secondary Antibody, Alexa Fluor 594 (Thermo Fisher Scientific, Cat# A-21207, dilution 1/1000)
Donkey anti-Mouse IgG (H+L) highly Cross-Adsorbed Secondary Antibody, Alexa Fluor 594 (Thermo Fisher Scientific, Cat# A-21203, dilution 1/1000)
Donkey anti-Mouse IgG (H+L) highly Cross-Adsorbed Secondary Antibody, Alexa Fluor 488 (Thermo Fisher Scientific, Cat# A-21202, dilution 1/1000)

The antibodies for western blotting:
Mouse monoclonal anti-MYH3 (Santa Cruz Biotechnology, Cat# sc-376157, dilution 1/1000)
Rabbit monoclonal anti-GAPDH (D16H11) (Cell Signaling Technology, Cat# 5174, dilution 1/5000)
Horseradish peroxidase (HRP)-conjugated anti-mouse IgG (Cell Signaling Technology, Cat# 7076, dilution 1/10000)
Horseradish peroxidase (HRP)-conjugated anti-rabbit IgG (Beyotime, Cat# A0208, dilution 1/10000)

Validation

APC-conjugated anti-pig CD31 (https://www.bio-rad-antibodies.com/monoclonal/pig-porcine-cd31-antibody-lci-4-mca1746.html?f=purified)
Alexa Fluor 647 anti-pig CD45 (https://www.bio-rad-antibodies.com/monoclonal/pig-porcine-cd45-antibody-k252-1e4-mca1222.html?f=alexa%20fluor%C2%AE%20647)
PE-conjugated anti-human CD56 (https://www.biolegend.com/en-us/products/pe-anti-human-cd56-ncam-antibody-1605)
Mouse monoclonal anti-Oct-3/4 (https://www.scbt.com/p/oct-3-4-antibody-c-10?requestFrom=search)
Mouse monoclonal anti-Sox2 (https://www.scbt.com/p/sox-2-antibody-e-4?requestFrom=search)

Rabbit polyclonal anti-human Nanog (https://www.peprotech.com/zh/anti-human-nanog)
Rabbit polyclonal anti-neuron specific beta III Tubulin (https://www.abcam.cn/products/primary-antibodies/beta-iii-tubulin-antibody-neuronal-marker-ab18207.html)
Rabbit polyclonal anti-alpha smooth muscle Actin (https://www.abcam.cn/products/primary-antibodies/alpha-smooth-muscle-actin-antibody-ab5694.html)
Rabbit monoclonal anti-Vimentin (https://www.abcam.cn/products/primary-antibodies/vimentin-antibody-epr3776-cytoskeleton-marker-ab92547.html)
Goat polyclonal anti-Brachyury (https://www.scbt.com/p/brachyury-antibody-n-19?requestFrom=search)
Rabbit monoclonal anti-human-γH2A.X (https://www.cellsignal.cn/products/primary-antibodies/phospho-histone-h2a-x-ser139-20e3-rabbit-mab/9718?site-search-type=Products&N=4294956287&Ntt=9718&fromPage=plp&_requestid=2648986)
Mouse monoclonal anti-PAX7 (https://dshb.biology.uiowa.edu/PAX7)
Rabbit polyclonal anti-MYOD1 (https://www.ptgcn.com/products/MYOD1-Antibody-18943-1-AP.htm)
Mouse monoclonal anti-Skeletal Myosin (Fast) (https://www.sigmaaldrich.cn/CN/zh/product/sigma/m4276)
Mouse monoclonal anti-Myosin heavy chain (MyHc) (https://dshb.biology.uiowa.edu/MF-20)
Actin-Tracker Red-594 (https://www.beyotime.com/product/C2205S.htm)
Donkey anti-Rabbit IgG (H+L) highly Cross-Adsorbed Secondary Antibody, Alexa Fluor 594 (https://www.thermofisher.cn/cn/zh/antibody/product/Donkey-anti-Rabbit-IgG-H-L-Highly-Cross-Adsorbed-Secondary-Antibody-Polyclonal/A-21207)
Donkey anti-Mouse IgG (H+L) highly Cross-Adsorbed Secondary Antibody, Alexa Fluor 594 (https://www.thermofisher.cn/cn/zh/antibody/product/Donkey-anti-Mouse-IgG-H-L-Highly-Cross-Adsorbed-Secondary-Antibody-Polyclonal/A-21203)
Donkey anti-Mouse IgG (H+L) highly Cross-Adsorbed Secondary Antibody, Alexa Fluor 488 (https://www.thermofisher.cn/cn/zh/antibody/product/Donkey-anti-Mouse-IgG-H-L-Highly-Cross-Adsorbed-Secondary-Antibody-Polyclonal/A-21202)
Mouse monoclonal anti-MYH3 (https://www.scbt.com/p/myh-antibody-b-5?requestFrom=search)
Rabbit monoclonal anti-GAPDH (D16H11) (Cell Signaling Technology, Cat# 5174, dilution 1/5000)
Horseradish peroxidase (HRP)-conjugated anti-mouse IgG (Cell Signaling Technology, Cat# 7076, dilution 1/10000)
Horseradish peroxidase (HRP)-conjugated anti-rabbit IgG (https://www.beyotime.com/product/A0208.htm)

Eukaryotic cell lines

Policy information about cell lines and Sex and Gender in Research

Cell line source(s)	The pgEpiSCs were derived from the embryo of NongDa Xiang pig in our previous work (Zhi et al., 2022, https://doi.org/10.1038/s41422-021-00592-9).<br>The mouse myoblast (C2C12) were purchased from Procell (CL-0044).<br>The porcine muscle stem cell line was isolated in this study and described within.
Authentication	The pgEpiSCs were authenticated by whole genome sequencing.<br>STR analysis was used for C2C12 authentication.<br>The porcine muscle stem cells authenticated by immunofluorescence staining.
Mycoplasma contamination	We confirmed that all cell lines tested were negative for mycoplasma contamination.
Commonly misidentified lines (See ICLAC register)	Commonly misidentified cell lines were not used in the study.

Animals and other research organisms

Policy information about studies involving animals; ARRIVE guidelines recommended for reporting animal research, and Sex and Gender in Research

Laboratory animals	The Kunming white mice (8-weeks-old) were used in the experiments were purchased from Beijing SiPeiFu Biotechnology Co., Ltd (Beijing, China) and used for the isolation of mouse embryonic fibroblasts (MEFs) for pgEpiSCs culture. All mice were individually housed under a 12hr light/dark cycle in a sterile environment and provided with food and water ad libitum.<br>The Nongda Xiang pigs (1-week-old) were used for the isolation of porcine muscle stem cells.
Wild animals	No wild animals were used in this study.
Reporting on sex	This study did not involve sex- and gender-based analysis because female and male mice were only used to isolate mouse embryonic fibroblasts (MEFs).
Field-collected samples	No field collected samples were used in the study.
Ethics oversight	All of the mouse and pig experiments performed were approved by the Institutional Animal Care and Use Committee of China Agricultural University.

Note that full information on the approval of the study protocol must also be provided in the manuscript.

Flow Cytometry

Plots

Confirm that:

- [x] The axis labels state the marker and fluorochrome used (e.g. CD4-FITC).
- [x] The axis scales are clearly visible. Include numbers along axes only for bottom left plot of group (a 'group' is an analysis of identical markers).
- [x] All plots are contour plots with outliers or pseudocolor plots.
- [x] A numerical value for number of cells or percentage (with statistics) is provided.

Methodology

Sample preparation	(1). The cells (pgEpiSCs-MPCs) were digested by TryPLE for 5 min into single cells and centrifuged at 1000 rpm for 5 min, which were washed three times with DPBS before being resuspended with diluted antibodies (Alexa Fluor 647 anti-pig CD45, APC-conjugated anti-pig CD31, PE-conjugated anti-human CD56) and incubated on ice for 30 min. Followed by washing three times with cold DPBS and resuspending precipitates for flow analysis of CD31- CD45-CD56+ ratios, where unstained cells were used as negative controls for delineation of FACS gating parameters.<br>(2).The mouse myoblast (C2C12), porcine muscle stem cells or pgEpiSC-MCs were cultivated on scaffolds and cell survival status was measured by using the Calcein / PI Cell Activity and Cytotoxicity Assay Kit. Cells were stained with Calcein-AM (AM) and propidium iodide (PI) double fluorescence, observed with a fluorescence microscope, and flow cytometry was used to determine cell survival effectiveness, where unstained cells were used as negative controls for delineation of FACS gating parameters.
Instrument	BD FACSVerse (BD Biosciences, USA).
Software	The FlowJo software was used in FACS data analysis.
Cell population abundance	The CD31-CD45-CD56+ population was defined as pgEpiSC-MPCs.<br>The Calcein-AM (AM, green) is characterized as a living cells.<br>The ropidium iodide (PI, red) is characterized as dead cells.
Gating strategy	The gating strategy is exemplified in Fig. 2 and Supplementary Fig. 5, 9, 11.The detailed method is shown in the method section (Flow cytometric analysis, Assay of cell survival efficiency on 3D edible scaffolds.).

- [x] Tick this box to confirm that a figure exemplifying the gating strategy is provided in the Supplementary Information.
